# The selenoprotein P–LRP5/6–WNT3A complex promotes tumorigenesis in sporadic colorectal cancer

**DOI:** 10.1172/JCI171885

**Published:** 2023-07-03

**Authors:** K. Sandeep Prabhu

**Affiliations:** Department of Veterinary and Biomedical Sciences, The Pennsylvania State University, University Park, Pennsylvania, USA.

## Abstract

Some studies suggest that the trace element selenium protects against colorectal cancer (CRC). However, the contribution of selenoprotein P (SELENOP), a unique selenocysteine-containing protein, to sporadic colorectal carcinogenesis challenges this paradigm. SELENOP is predominately secreted by the liver but is also expressed in various cells of the small intestine and colon in mice and humans. In this issue of the *JCI*, Pilat et al. demonstrate that increased SELENOP expression promoted the progression of conventional adenomas to carcinoma. SELENOP functioned as a modulator of canonical WNT signaling activity through interactions with WNT3A and its coreceptor LDL receptor–related protein 5/6 (LRP5/6). Secreted SELENOP formed a concentration gradient along the gut crypt axis, which might amplify WNT signaling activity by binding to LRPL5/6. The mechanism for WNT control via SELENOP may affect colorectal tumorigenesis and provide therapeutic targets for CRC.

## Selenoproteins and intestinal tumorigenesis

Selenium is an essential trace element that is incorporated into several proteins (25 in humans and 24 in mice) as selenocysteine (Sec), which is also known as the 21st amino acid. Selenoprotein synthesis machinery requires Sec-tRNA^Sec^, a specialized elongation factor (EFSec), and Sec insertion sequence (SECIS) binding protein 2 (SBP2). SBP2 recognizes SECIS, which forms a unique secondary hairpin loop structure in the 3′-UTR of the mRNA, and cotranslationally incorporates Sec at the penultimate UGA codon (1). A rare heterozygous mutation within SBP2 was reportedly linked to selenoprotein deficiency and multisystem dysfunction, including ileocolonic inflammation (2). Studies in mouse models of azoxymethane-induced (AOM-induced) colon cancer and selenoprotein gene deletion studies further suggest that selenoproteins play a pivotal role in maintaining gut homeostasis (3). Selenoproteins, glutathione peroxidases (GPXs), selenoprotein F (SELENOF), and selenoprotein P (SePP1, also known as SELENOP) have been extensively studied for their role in chronic intestinal inflammation, including colorectal tumorigenesis (4–6). Whereas SELENOP is a plasma selenoprotein secreted by the liver and also expressed in cells of the small intestine and colon, GPX2 and SELENOF are intracellular proteins. Deletion of gastrointestinal glutathione peroxidase 2 (Gpx2), normally expressed at the crypt base, protects mice from AOM-induced inflammation CRC (7), while a similar reduction in aberrant crypt foci (ACF) is also seen in *SelenoF*-KO mice (5). Interestingly, this finding was also recapitulated in experiments in which selenoprotein expression was genetically impaired upon mutation of tRNA^Sec^ (3). However, heterozygous *SelenoP*-KO mice develop more ACFs upon AOM/dextran sodium sulfate (DSS) treatment, suggesting an opposite trend. Notably, in global *SelenoP*-KO mice, a decrease in tumor numbers has been attributed to exacerbation of oxidative stress and cytotoxicity (8). These studies also suggest the presence of a distinct SELENOP pool in the intestinal epithelial cells that could contribute to localized selenoprotein expression that perhaps affects tumorigenesis.

**Figure 1:**
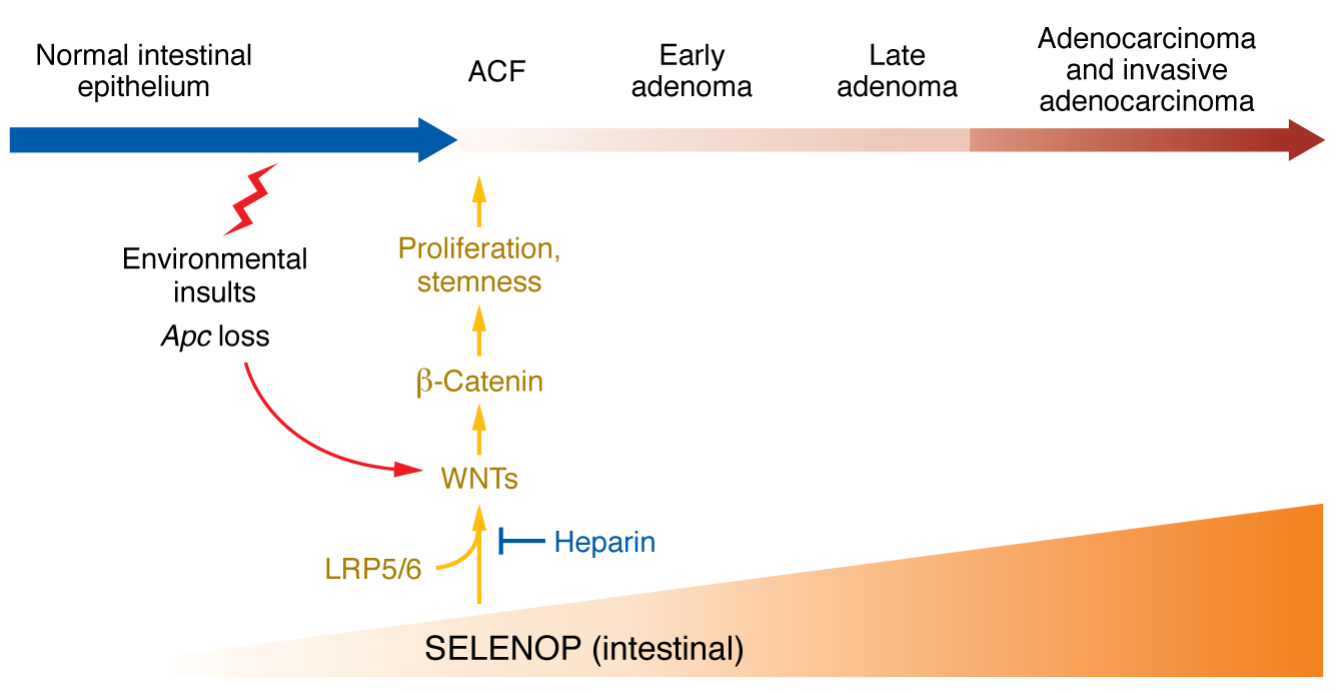
SELENOP increases canonical WNT signaling to promote *Apc*-dependent tumorigenesis in the intestine. Loss of the tumor suppressor gene *Apc* results in activation of WNT signaling. Increased expression of SELENOP throughout the progression from conventional adenomas to carcinoma drives carcinogenesis in sporadic CRC. A 42 amino acid domain in SELENOP situated between the third and fourth Sec residues activates canonical WNT signaling via direct interaction with WNT3A and its coreceptor LRP5/6 to increase β-catenin–dependent expression of protumorigenic genes. Notably, treatment with heparin can prevent the interaction between SELENOP and LRP5/6.

In this issue of the *JCI*, Pilat et al. conducted selenotranscriptome analyses of normal WT mouse small intestine and colon epithelial cells that corroborated the high levels of expression of *Selenop* (and a few other selenoproteins) seen in the Gut Cell Atlas scRNA-Seq of normal human colon and small intestine epithelium (9). Consistent with this observation, human small intestinal organoids (enteroids) indicated high levels of SELENOP expression, particularly in those enteroids that were differentiated toward enterocytes, goblet cells, or Paneth cells. Findings from the Cellular Trajectory Reconstruction Analysis Using Gene Counts and Expression (CytoTRACE) showed that *SELENOP* expression progressively increased from baseline in normal crypt stem cells to elevated levels in adenoma-specific cells and even higher levels in microsatellite-stable (MSS) cancers. These results suggest that upregulation of SELENOP expression throughout conventional CRC is a function of stemness. Furthermore, crossing *Selenop-*KO mice (maintained on a selenium-supplemented diet) with a mouse model that mimics loss of the tumor suppressor gene adenomatous polyposis coli (*Apc*), as in human CRC, decreased colon tumor incidence and area in the mice, despite similar survival and dysplasia severity. Clearly, these data indicate a tumor-promotive role for SELENOP in *Apc*-dependent tumorigenesis (Figure 1).

SELENOP, beyond serving as a protein involved in selenium-transport from the liver to extrahepatic sites, takes up selenium from the plasma or from locally secreted sources in the gut through endocytosis by LDL receptor–related proteins (LRPs) (10). LRP1, LRP2 (Megalin), and LRP8 (ApoER2), are expressed in a tissue- and cell-specific manner. Interestingly, LRP1, -2, and -8 are not expressed at high levels in the small intestine and colon, suggesting the likelihood that other proteins serve the same function. On the basis of these data, Pilat and colleagues postulated that LRP5 and -6 (LRP5/6), which share approximately 70% sequence identity, could serve as receptors for SELENOP in the gut. Elegant use of a proximity ligation assay in 293T cells along with classical immunoprecipitation experiments confirmed the interaction between SELENOP and LRP6. Furthermore, Pilat et al. meticulously mapped the interaction domain to the area between the third and fourth Secs in SELENOP. This domain was critical in the interaction with either LRP5 or LRP6. The authors proposed that such an interaction would internalize SELENOP to amplify the WNT signaling activity in human CRC tumoroids. Notably, cells treated with purified human SELENOP protein increased WNT-dependent target gene expression, thus supporting this theory. Furthermore, SELENOP overexpression rescued the tumoroid-forming capacity and WNT target gene expression in SELENOP-deficient tumoroids (9).

## The SELENOP and WNT pathway in CRC

The WNT pathway is composed of a family of proteins that not only play critical roles in embryonic development and adult tissue homeostasis but, upon deregulation, can lead to serious diseases, including cancer. The canonical WNT pathway controls cell proliferation through the nuclear translocation of β-catenin to activate T cell factor/lymphoid enhancer–binding transcription factor (TCF/LEF) and its downstream target genes. In the presence of WNT proteins, frizzled (FZD) and LRP5/6 recruit the destruction complex comprising APC, glycogen synthase kinase 3 protein (GSK3), the scaffold protein AXIN, and casein kinase 1 (CK1) to inhibit the degradation of β-catenin. In contrast, this complex facilitates the degradation of β-catenin in the absence of WNT proteins. Mutations in APC seen in the majority of colorectal adenomas and CRC, or deletion of the APC allele, inhibit the formation of the degradation complex, which activates β-catenin and increases the self-renewal potential of cancer cells to drive CRC development. Pilat et al. reported the effect of *Selenop* KO on adenomas, and not ACF, which is more commonly used as a readout in experimental tumorigenesis (9). That said, previous studies in colitis-associated carcinoma (CAC) models showed dysregulated WNT signaling in *Selenop*-KO tumors that corroborates with the current studies using *Apc^Δ/E/+^* *Selenop^–/–^* tumoroids, thus emphasizing the importance of SELENOP in WNT signaling in both sporadic CRC and chemically-induced CAC models (11, 12). Furthermore, Pilat and co-authors suggest the presence of a *SELENOP* expression gradient from the crypt base to the top of the normal colon, as well as a pattern of *SELENOP* expression that continues to increase with tumor severity, from tumor-initiating stem cells to adenomatous polyps, reaching its highest levels in MSS cancers (9). Such a possibility highlights the importance of stratifying *SELENOP* expression by epithelial cell type and suggests that the mechanisms that control its increased expression are key factors in the development of CRC.

Pilat and co-workers also describe an interesting mechanism of fine-tuning of WNT signaling by SELENOP that involves cell-surface glycoproteins, such as heparin sulfate glycoproteins (HSPGs) (9). HSPGs represent either cell-surface or extracellular matrix (ECM) molecules with one or more heparan sulfate (HS) chains covalently bound to the protein backbone. Pilat et al. report that HSPGs inhibited the interaction of SELENOP with LRP6, but not LRP8. However, inhibition of HSPGs increased the interaction of SELENOP with LRP6 (9). This finding is particularly intriguing, since SELENOP interacts with heparin through putative histidine- and lysine-rich sequences in a pH-dependent manner (13). Thus, the expression of HSPGs and modulation of localized pH along the gut crypt axis or during various stages of CRC could affect the SELENOP-dependent WNT signaling mechanisms in addition to ensuring some level of cell type–specific uptake and function of SELENOP.

## Conclusions

Pilat et al. (9) deepen our understanding of WNT signaling in sporadic CRC via the interaction of SELENOP with LRP5/6, in addition to LRP1, LPR2, and LRP8. An interesting follow up that correlates the expression of LRP5/6 with that of WNT3A and SELENOP in the small intestine and colon in normal and CRC samples could determine whether the upregulation of SELENOP expression throughout conventional carcinogenesis is a function of stemness. Such an effort, combined with profiling of other selenoproteins, could provide information regarding the ability of SELENOP in potential temporal mechanisms of redox control in epithelial and other cell types of the gastrointestinal tract during tumorigenesis. Another intriguing mechanism that could be at the heart of the control of WNT signaling is HSPG-mediated SELENOP-LRP5/6 interactions, in particular, which may determine SELENOP-WNT3A interaction and activation. The interaction of HSPGs in SELENOP-dependent WNT signaling could be further gleaned from the enteroid and tumoroid models. HSPGs, such as syndecans and perlecans, are upregulated in colon cancer to increase cell migration, proliferation, and metastasis (14). However, their activity is also under the control of endosulfatases (SULFs) that can trim the heparin side chains, which may ultimately control the activity of SELENOP-dependent WNT signaling, making HSPGs and SULFs potential therapeutic targets.

Interestingly, SNPs in human *SELENOP* can result in varied expression of selenoproteins in response to the consumption of selenium-rich foods (15). Thus, it is important to extend these studies in the context of selenium nutrition to assess the effects of selenium supplementation on patients with CRC. The Pilat et al. (9) results add another layer of complexity to the existing and diverse mechanisms of WNT signaling, particularly given the pro-tumorigenic role of SELENOP.

## Version 1 07/03/2023

Electronic publication
